# Ocular Syphilis as a Primary Manifestation of Neurosyphilis: A Case Report

**DOI:** 10.7759/cureus.59651

**Published:** 2024-05-04

**Authors:** Rosaida Silverio Lopez, Venkata Gandi, Syed Askari Hasan

**Affiliations:** 1 Internal Medicine, South Georgia Medical Center, Valdosta, USA

**Keywords:** uncommon sti presentation, temporary vision loss, rpr, sexually transmitted infection, neurosyphilis, ocular syphilis

## Abstract

Sexually transmitted infectious diseases could affect a variety of organs, generating significant symptomatology. In the elderly population, infectious causes for vision problems are not generally considered. We present the case of an elderly patient with blurred vision and darkening of visual fields. He underwent an unsuccessful biopsy of the temporal artery as his vision disturbances presented also with episodic headaches. He was found to have an elevated rapid plasma reagin (RPR) and venereal disease research laboratory (VDRL) test in his cerebrospinal fluid (CSF) analysis. He was treated for ocular syphilis with a total resolution of his vision loss.

## Introduction

The incidence of sexually transmitted infections (STIs) has almost doubled among US adults aged 65 and older in the last few decades [1]. This increase could be caused by misperceptions and lack of information among the elderly population when it comes to prevalent STIs including chlamydia, gonorrhea, syphilis, and human immunodeficiency virus (HIV). Current Centers for Disease Control and Prevention (CDC) and the United States Preventive Services Task Force (USPSTF) guidelines are limited in the recommendations for STI testing in the elderly population, and most current guidelines utilize the age maximum of 65 years as a limit age for certain STI screening [2]. The presented case report highlights the importance of the evaluation for possible infectious causes in common advanced age complaints including vision loss, presbycusis, and gait disturbances, among others.

## Case presentation

The patient is a 68-year-old male with a past medical history of coronary artery disease, status post coronary artery bypass grafting (CABG), hypertension, hyperlipidemia, and previous verrucous vocal cord carcinoma, treated with radiation, now in remission, who was sexually active in a monogamous heterosexual relationship, with no history of syphilitic chancre or other STIs, and who complained of a three-month history of painless vision loss. 

An ophthalmology specialist saw him in an outpatient setting. His initial symptoms and vision exam findings (see Table 1) raised concern for a combined form of age-related cataract and presbyopia. He was prescribed Visine Tears 0.2%-0.2%-1% one drop as needed in both eyes.

**Table 1:** Comprehensive vision assessment DSC: distant chart with correctors; RAPD: relative afferent pupillary defect; OCT: optical coherence tomography; RNFL: retinal nerve fiber layer

Assessment	Right eye	Left eye
Visual acuity	DSC 20/25	DSC 20/40-1
Intraocular pressure measured with ICare	15	17
External eye exam	Findings	Findings
Pupils	Round. Brisk. No RAPD. 4 millimeters	Round. Brisk. No RAPD. 4 millimeters
Motility	Full. Orthotropic	Full. Orthotropic
CVF	Full	Full
Adnexa	Normal ocular adnexa	Normal ocular adnexa
Anterior eye exam	Findings	Findings
Lids	Lids normal	Lids normal
Cornea	Epithelium intact. Clear stroma. Clear endothelium	Epithelium intact. Clear stroma. Clear endothelium
Anterior chamber	Normal depth. Quiet	Normal depth. Quiet
Iris	Flat	Flat
Lens	No posterior subcapsular cataract, nuclear sclerosis	No posterior subcapsular cataract, nuclear sclerosis
Posterior eye exam	Findings	Findings
Nerve	Disc edema. Disc hemorrhage	Disc edema
Vitreous	Clear	Clear
Retinal vessels	Normal caliber	Normal caliber
Macula	No edema	No edema
Periphery	No holes or tears. Attached 360 degrees	No holes or tears. Attached 360 degrees
Fundus photos findings	Mild disc edema. Normal retinal vessels. Flame-shaped hemorrhages	Mild disc edema. Normal retinal vessels
OCT/RNFL	Ocular nerve edema	Ocular nerve edema

Repeat evaluation a month later showed unimproved vision findings (see Table 2), and a diagnosis of anterior ischemic optic neuropathy was established (right eye>left eye). A possible diagnosis of giant cell arteritis (GCA) was also considered, as his erythrocyte sedimentation rate levels were high, and he had sporadic headaches along with vision loss. He received oral prednisone 60 milligrams daily for two weeks with subsequent improvement in his vision acuity. This dose was tapered to 40 milligrams daily, and his symptoms recurred shortly. A temporal artery biopsy returned negative, and although a negative biopsy does not completely rule out GCA due to skip lesions, this diagnosis was less probable. His mental status was described as appropriate, and he showed normal mood/affect during all of his clinic visits. 

**Table 2:** Comprehensive vision assessment one month after initial outpatient presentation DSC: distant chart with correctors; PHNI: pinhole no improvement; RAPD: relative afferent pupillary defect; CDR: cup-to-disc ratio

Assessment	Right eye	Left eye
Visual acuity	DSC 20/50. PHNI	DSC 20/30+1
Intraocular pressure measured with ICare	13	12
Color plates	3 out of 14 plates correct	13 out of 14 plates correct
External eye exam	Findings	Findings
Pupils	Round. Brisk. No RAPD. 4 millimeters	Round. Brisk. No RAPD. 4 millimeters
Motility	Full. Orthotropic	Full. Orthotropic
CVF	Full	Full
Adnexa	Normal ocular adnexa	Normal ocular adnexa
Anterior eye exam	Findings	Findings
Lids	Lids normal	Lids normal
Cornea	Epithelium intact. Clear stroma. Clear endothelium	Epithelium intact. Clear stroma. Clear endothelium
Anterior chamber	Normal depth. Quiet	Normal depth. Quiet
Iris	Flat	Flat
Lens	No posterior subcapsular cataract. Nuclear sclerosis. cortical	No posterior subcapsular cataract, nuclear sclerosis. Cortical
Posterior eye exam	Findings	Findings
Nerve	Mild disc edema. No disc hemorrhage. CDR <0.1	CDR 0.1 slightly elevated, crowded, no pallor? small sub heme
Vitreous	Clear	Clear
Retinal vessels	Normal caliber	Normal caliber
Macula	No edema	No edema
Periphery	No holes or tears. Attached 360 degrees	No holes or tears. Attached 360 degrees
24-2 Humphrey visual field test results	Paracentral loss	Increased blind spot

His steroid started to be tapered, which worsened his visual acuity with a perceived darkening and blurring of vision bilaterally and in all vision fields, requiring hospitalization for further investigation. He denied diplopia, weakness, numbness, tingling, facial paralysis, decreased motor strength, or reduced sensation in the upper or lower extremities. During physical examination, his vital signs were normal. He was not in acute distress. He had no behavioral disturbances or psychiatric symptoms. No facial asymmetry was noticed. Extraocular movements were intact. He had decreased visual acuity on the Snellen chart at 6 feet and defective color differentiation (see Table 3). His upper and lower extremity motor and sensory function was preserved.

**Table 3:** Visual acuity results during hospitalization

Content	OD (right eye)	OS (left eye)	OU (both eyes)	Reference range
Uncorrected	20/70	20/200	20/50-2	20/20
Corrected	20/50	20/200	20/50	20/20
Color differentiation	Deficits with green versus blue			No deficits

**Figure 1:**
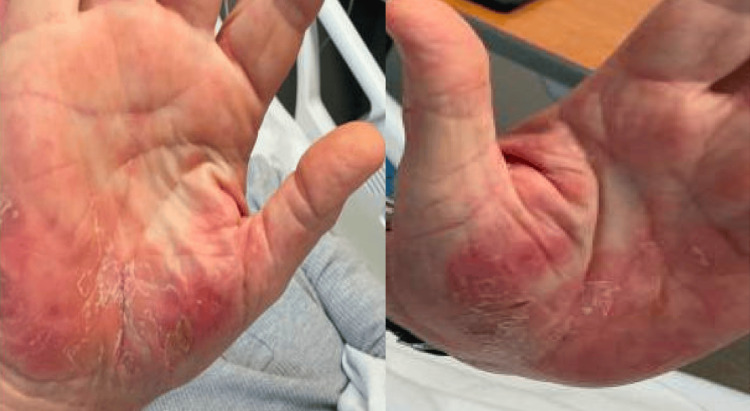
Rash present in palms bilaterally

The palms and soles presented a desquamating rash with erythema and tenderness to the touch (see Figure 1). He had no genital rash or penile discharge. 

Investigations

On admission, laboratory studies showed various abnormalities, including an elevated white blood cell (WBC) count of 17.00 10^3^/microliter and an erythrocyte sedimentation rate of 42 millimeters/hour. A rapid plasma reagin (RPR) test was positive at 1:1024. A fluorescent treponemal antibody absorption test was reactive (see Table 4, Table 5, and Table 6).

**Table 4:** Complete blood count on hospital admission WBC: white blood cell; RBC: red blood cell

Content	Result	Reference range and units
WBC count	17.00	4.80-10.80 10^3^/microliter
RBC count	4.32	4.70-6.10 10^6^/microliter
Hemoglobin	12.9	14.0-18.0 gram/deciliter
Hematocrit	38.1	42.0-52.0%
Mean corpuscular volume	88.3	78.0-98.0 femtoliter
Mean corpuscular hemoglobin	30.0	27.0-31.0 picogram
Mean corpuscular hemoglobin concentration	33.9	33.0-37.0 gram/deciliter
Red cell distribution width	17.0	11.5-14.5%
Platelets	269	130-400 10^3^/microliter
Mean platelet volume	6.8	7.4-10.4 femtoliter

**Table 5:** Basic metabolic panel on hospital admission

Content	Result	Reference range and units
Sodium	135	135-148 millimole/liter
Potassium	4.2	3.5-5.0 millimole/liter
Chloride	98	98-109 millimole/liter
CO2	30	22-31 millimole/liter
Anion gap	7	7-16 millimole/liter
Glucose	87	70-100 milligram/deciliter
Blood urea nitrogen	29	8-20 milligram/deciliter
Creatinine	0.70	0.70-1.30 milligram/deciliter
Calcium	9.0	8.9-10.3 milligram/deciliter
Estimated glomerular filtration rate	>60.0	>60.0 milliliter/minute/1.73meter^2^

**Table 6:** Inflammatory markers and other laboratory studies RPR: rapid plasma reagin; FTA-ABS: fluorescent treponemal antibody absorption

Content	Result	Reference range and units
C-reactive protein	4.0	≤10.0 milligram/liter
Erythrocyte sedimentation rate	42	0-20 millimeter/hour
Prothrombin time	11.4	9.4-12.5 seconds
International normalized ratio	1.00	0.8-1.1
Partial thromboplastin time	20.9	25.1-36.5 seconds
Antinuclear antibody	0.6	≤1.0 units
c-Antineutrophilic cytoplasmic antibody	Negative	Negative
p-Antineutrophilic cytoplasmic antibody	Negative	Negative
RPR	Reactive 1:1024	Non-reactive
FTA-ABS test	Reactive	Non-reactive
Human immunodeficiency virus combo	Negative	Negative

**Figure 2:**
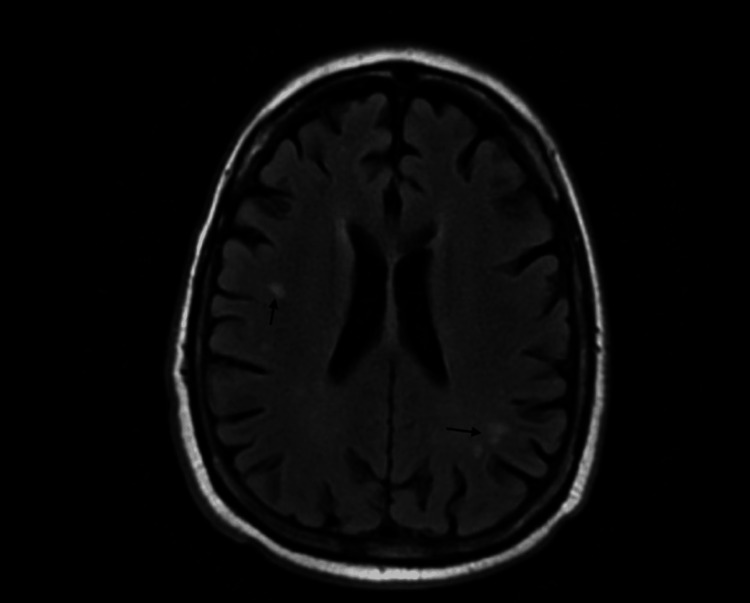
White matter changes in brain MRI MRI: magnetic resonance imaging

**Figure 3:**
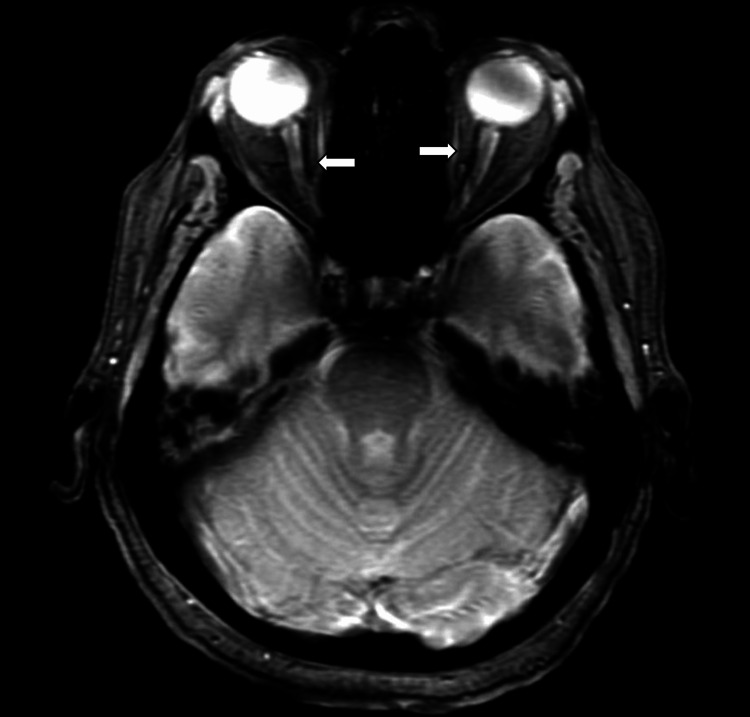
Orbit MRI showing increased CSF MRI: magnetic resonance imaging; CSF: cerebrospinal fluid

Computed tomography angiography (CTA) of the neck showed chronic 100% occlusion of the right internal carotid artery with appropriate collaterals. Magnetic resonance image (MRI) of the orbit/face/neck with and without contrast showed white matter changes in the brain (see Figure 2) and mildly increased cerebrospinal fluid (CSF) surrounding the optic nerves bilaterally (see Figure 3). A lumbar puncture was performed to evaluate intracranial CSF pressure and due to concern of neurosyphilis in a patient with ocular symptoms and positive syphilis screening studies. A meningitis panel and CSF studies on malignancy were negative. VDRL in the CSF was positive, confirming the diagnosis of neurosyphilis with optic neuropathy. Other significant findings in CSF studies included an elevated WBC, glucose, and protein (see Table 7). 

**Table 7:** Spinal fluid analysis CSF: cerebrospinal fluid; RBC: red blood cell; WBC: white blood cell; VDRL: venereal disease research laboratory

Content	Result	Reference range
Opening pressure	19	10-20 cm H2O
Protein, CSF	77	15-45 milligram/deciliter
Glucose, CSF	85	40-70 milligram/deciliter
Color	All colorless	Colorless
Clarity	All clear	Clear
RBC	6	≤0
WBC	19	0-5
VDRL	Positive	Negative

Treatment

The Centers for Disease Control and Prevention (CDC) treatment recommendation for ocular syphilis is 18-24 million units of aqueous crystalline penicillin G per day. This medication should be administered through intravenous injections of 3-4 million units every four hours or continuous infusion for 10-14 days [3]. In cases where compliance is not an issue, an alternative approach of procaine penicillin G at a daily dose of 2.4 million units, accompanied by probenecid 500 mg four times daily, can be considered for 10-14 days. If an individual has a penicillin allergy, it is advisable to undergo penicillin desensitization [3]. Our patient was administered penicillin G potassium 4 million units every four hours for 14 days.

Outcome and follow-up

Upon discharge from the hospital, the patient was followed up by an infectious disease doctor. Ten months later, his inpatient care team contacted him. He reported that his vision disturbances had resolved, and he returned to full functionality in daily activities, such as driving. Unfortunately, the patient did not follow up with ophthalmology. 

## Discussion

Neurosyphilis is an infection caused by *Treponema pallidum* species of the central nervous system. Ocular syphilis falls under the category of neurosyphilis. It can lead to vision loss [4].

The presentation of syphilis varies across its different stages. Ocular syphilis can manifest at any point during the progression of the disease. Primary syphilis is linked to the initial contact with the *T. pallidum* bacterium at the site of infection. In cases of primary syphilis, ocular symptoms may include the development of chancres on the eyelids and conjunctiva due to direct exposure to bodily fluids and contaminated hands [5-8].

If primary syphilis is left untreated, it can progress to secondary syphilis, potentially affecting the palms of the hands and the soles of the feet. This stage can lead to a rash that may also involve the eyelids and result in conditions such as blepharitis, madarosis, conjunctivitis, scleritis, keratitis, and iridocyclitis. Additionally, around 1-2% of patients with secondary syphilis may experience acute meningitis, which can cause increased intracranial pressure and papilledema. Other ophthalmic manifestations of secondary syphilis encompass optic neuritis, optic neuropathy, and the presence of Argyll Robertson pupils [5-8].

In contrast, tertiary syphilis is characterized by the formation of gummas and a worsening neurological status. Gummas are chronic granulomatous lesions that can lead to scarring and may develop on the skin, potentially involving the eyelids as well [5-8].

In a case report published in March 2022, a 57-year-old male presented with eye pain and was sent home after receiving prednisolone 60 milligrams per day due to positivity for autoimmune diseases [9]. However, the patient was not compliant with the steroid therapy. A week later, the patient was reevaluated for no improvement of his ocular symptoms and underwent a workup for neurosyphilis with VDRL and IgG and IgM treponemal tests, which ended up being positive. Compared to this 57-year-old male, our patient was suffering from loss of visual acuity for three months, which shows that the diagnosis of ocular syphilis is often delayed.

In a retrospective analysis of 17 cases of ocular syphilis in Suzhou, China, seven patients were misdiagnosed with other causes of visual acuity impairment [10]. The use of symptomatic medication for ocular inflammation, such as steroids, was deemed to be an important cause of misdiagnosis in this investigation [10]. Similarly, our patient was suffering from vision loss and was initially misdiagnosed with the suspicion of GCA for which he received steroids with temporary improvement of his symptoms. In another study, a total of 8310 new cases of syphilis were seen, of which 213 patients had ocular disease and 50 patients had blindness due to syphilis [11]. Increasing age and higher RPR titers were associated with ocular involvement. Fifty patients (and a total of 67 eyes) met the WHO definition of blindness before treatment for syphilis. At the end of follow‐up, vision had improved in 24 of 67 eyes (35.8%) after treatment. A crucial point illustrated with the comparison of these studies to our case is that ocular syphilis may be missed due to mimicking inflammatory conditions and reflects the need to test for syphilis in individuals with loss of visual acuity to avoid permanent vision damage as prompt treatment can reverse the symptoms. 

In a systematic review of diagnostic tools for neurosyphilis in 2021, CSF-VDRL and CSF-RPR were gold standards for confirming diagnosis in most studies [12]. However, both tests are operator-dependent. CSF treponemal tests are more specific but less sensitive [12]. As a result, low sensitivity of CSF-VDRL does not rule out disease when negative. In our patient, CSF-VDRL was positive, and thus, a diagnosis of neurosyphilis was achieved. The problem lies when CSF-VDRL is negative and neurosyphilis is still suspected. More sensitive tests need to be explored to prevent permanent disability from neurosyphilis due to a negative test. 

## Conclusions

When assessing for neurological findings in the elderly population, it is important to consider infectious etiologies including STIs. Visual acuity loss could be an indicator of ocular syphilis. Individuals who exhibit these symptoms should be screened for syphilis. Early detection of ocular syphilis and adherence to medical treatment are critical to avoiding irreversible vision impairment.

A more sensitive test for neurosyphilis needs to be developed, which would lead to less invasive procedures. Perhaps, establishing lumbar puncture as part of the standard of care for vision loss in the elderly population could modify care for this age group, as advanced age showed a worse prognosis in ocular syphilis.

The diagnosis of ocular syphilis is based on clinical symptoms, RPR, VDRL, and ocular examination. In our case, CSF analysis allowed a diagnosis to be confirmed months after the patient's start of symptoms, and his clinical presentation improved. Regardless of the time elapsed since the suspicion of ocular syphilis, consideration for further testing is important for proper diagnosis and treatment. 
